# A Case of Apoplexy of Rathke's Cleft Cyst Followed by Cerebral Infarction

**DOI:** 10.1155/2015/645370

**Published:** 2015-02-25

**Authors:** Yu-ichiro Ohnishi, Yasunori Fujimoto, Koichi Iwatsuki, Toshiki Yoshimine

**Affiliations:** ^1^Department of Neurosurgery, Osaka University Medical School, Suita, Osaka 565-0871, Japan; ^2^Department of Neurosurgery, Osaka Neurological Institute, Toyonaka, Osaka 565-0871, Japan

## Abstract

Rathke's cleft cyst (RCC) apoplexy is a rare clinical entity. We report a case of apoplexy of an RCC followed by cerebral infarction. A 67-year-old woman was found lying on the street unconscious. She had fallen from her motorbike. On referral to our hospital she gradually regained consciousness and presented with no neurological deficits. CT showed a round and slightly hyperdense area in the suprasellar region. However, the attending physician did not find this abnormal finding on CT and the patient was discharged the same day. Thirteen days after the first emergency visit she developed left hemiparesis and dysarthria. CT showed a round hypodense area in the suprasellar region. The change of the density in the suprasellar region on CT suggested the pituitary apoplexy. CT also showed a low density area in the territory of the right middle cerebral artery, which indicated the cerebral infarction. MR angiography revealed poor visibility and stenotic changes of right middle cerebral arteries. Transsphenoidal surgery was performed. Histopathological findings confirmed a hemorrhagic RCC. Postoperative MR angiography showed that the visibility and stenosis of right middle cerebral arteries were recovered. This is the rare case of apoplexy of an RCC followed by cerebral infarction.

## 1. Introduction

Rathke's cleft cysts (RCCs) were found frequently (13–22%) in normal pituitary glands at autopsy [1]. Most RCCs are small and asymptomatic throughout life. RCC presents various symptoms when it enlarges to compress the optic chiasm, hypothalamus, and pituitary gland [2–4]. The symptoms include headache, visual disturbance, hypopituitarism, and diabetes insipidus. Apoplexy of an RCC is a very rare clinical entity. Pituitary apoplexy occurs in 0.4–16.6% of all pituitary adenomas [5, 6]. Pituitary apoplexy followed by cerebral infarction is rare. There are 2 possible mechanisms of cerebral infarction in pituitary apoplexy, that is, vasospasm or compression of an artery. This is the rare case of apoplexy of an RCC followed by cerebral infarction.

## 2. Case Report

**Figure 1:**
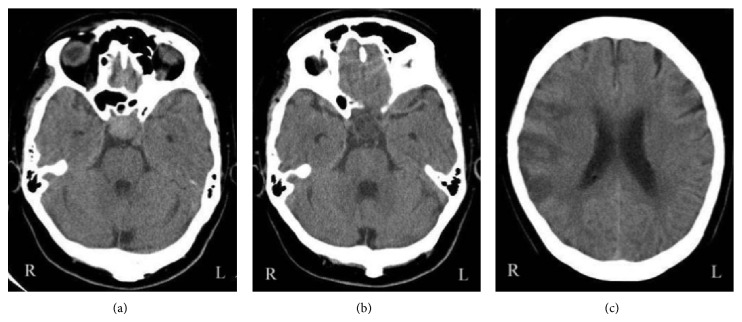
(a) Axial view of the CT showing the round and slightly hyperdense area in the suprasellar region at the first emergency visit. (b, c) Axial view of the CT showing the round hypodense area in the suprasellar region and the low density area in the territory of the right middle cerebral artery at the second emergency visit.

A 67-year-old woman was found lying on the street unconscious. She had fallen from her motorbike. On referral to our hospital she gradually regained consciousness and presented with no neurological deficits. She had a facial abrasion, but no headache and no nausea. CT showed a round and slightly hyperdense area in the suprasellar region (Figure 1(a)). However, the attending physician did not consult a neurosurgeon and the patient was discharged the same day. This physician overlooked a significant appearance in the suprasellar region.

Thirteen days after the emergency visit, she gradually developed left hemiparesis. Other neurological signs were within normal limits. Arterial blood gas measurement did not reveal acidemia or alkalemia. Routine laboratory tests were normal, and the patient was neither diabetic, nor hypertensive, nor hyperlipidemic. Endocrinological tests also detected no abnormalities. Echocardiographic examination and carotid artery ultrasound examination showed no abnormalities, and the electrocardiogram was also normal.

**Figure 2:**
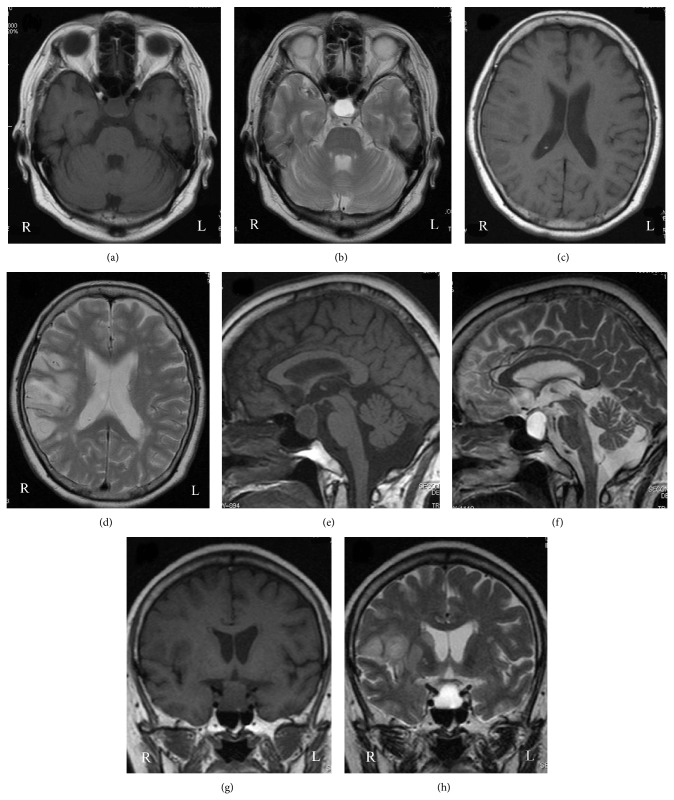
Axial, sagittal, and coronal views of the MRI showed isointensity on T1WI (a, e, and g) and high intensity on T2WI (b, f, and h) in the suprasellar region. Axial and coronal views of the MRI presented isolow intensity on T1WI (c, g) and high intensity on T2WI (d, h) in the territory of the right middle cerebral artery.

**Figure 3:**
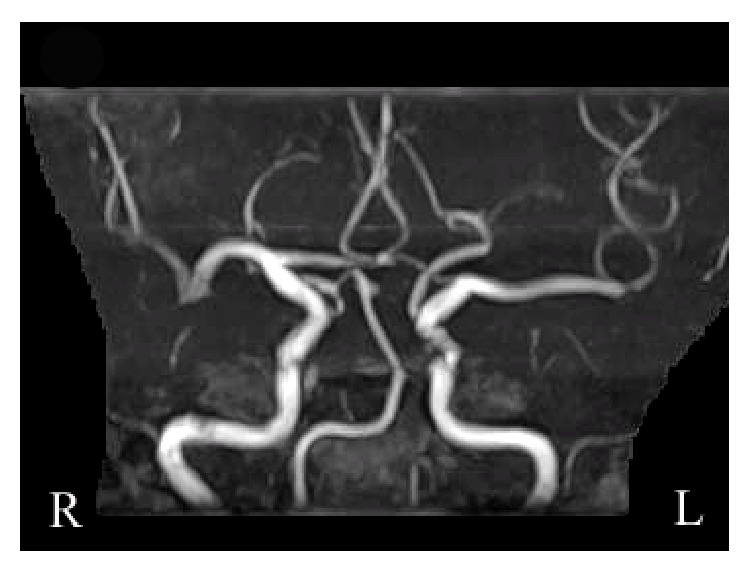
MRA revealed less visibility and stenotic changes of right middle cerebral arteries.

CT on admission showed a round hypodense area in the suprasellar region (Figure 1(b)). The change of the density in the suprasellar region on CT suggested the pituitary apoplexy. Magnetic resonance imaging (MRI) taken 2 days after admission detected isointensity on T1WI and high intensity on T2WI in the suprasellar region (Figure 2). CT also showed a low density area in the territory of the right MCA, which indicated the cerebral infarction (Figure 1(c)). MR angiography (MRA) showed signal loss with poor visibility of distal right middle cerebral arteries (MCAs) (Figure 3). The conservative treatment for cerebral infarction improved her left hemiparesis.

**Figure 4:**
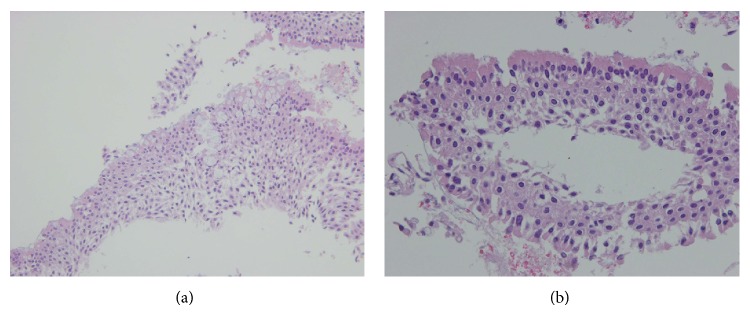
The cyst wall shows ciliated columnar epithelium with goblet cells. Thin blood vessels were observed in the cyst wall. Red blood cells were seen in both cyst and cyst wall (H&E original magnification: (a) ×200; (b) ×400).

**Figure 5:**
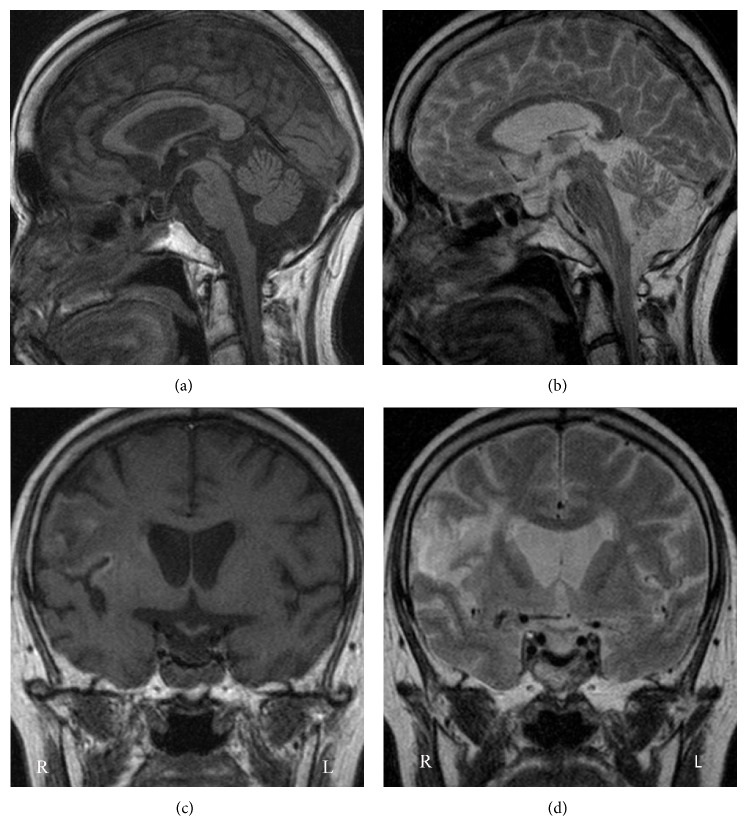
Sagittal and coronal views of the MRI showed the decompression of suprasellar region. (a, c) T1WI and (b, d) T2WI.

**Figure 6:**
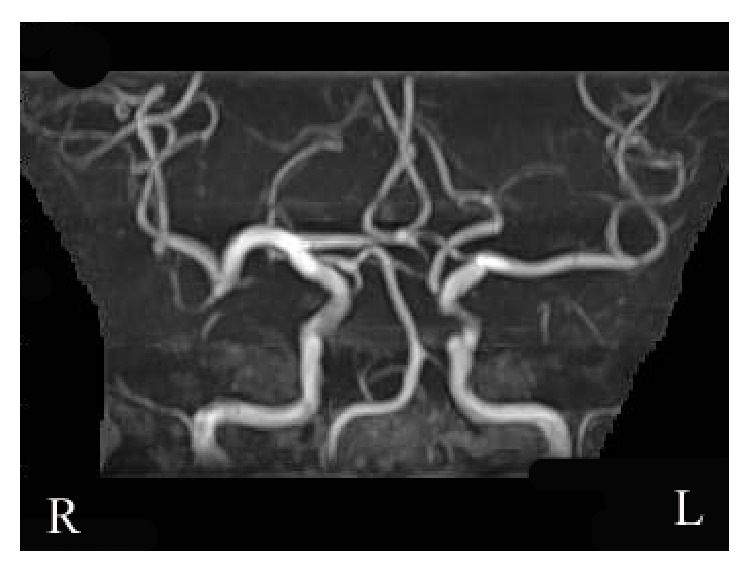
Postoperative MRA revealed partial stenotic changes of right MCAs.

Ten days after admission, endoscopic endonasal transsphenoidal surgery was performed. The sella turcica was found to be thin. The tumor contained white-tinged viscid fluid. A normal pituitary gland was observed on the right side. Histopathological findings confirmed hemorrhagic RCC (Figures 4(a) and 4(b)). The cyst wall was lined by a ciliated columnar cell layer with goblet cells. Thin blood vessels were observed in the cyst wall. Red blood cells were seen in both cyst and cyst wall. Postoperative MRI showed the decompression of suprasellar region (Figure 5). Postoperative MRA revealed partial stenotic changes of right MCAs (Figure 6). These findings suggested RCC apoplexy followed by cerebral infarction.

## 3. Discussion

RCC apoplexy is a rare clinical entity. Fourteen cases of apoplexy of RCC have been reported in the literature (Table 1) [6–17]. The mechanism of RCC apoplexy is supposed to be the repeated minor bleeding from the thin blood vessels in the cyst wall by the stimulation of cyst contents and the bleeding from the hypophyseal portal blood vessels by the compression or shearing stress [7, 15]. The clinical presentations of these cases were headache, nausea, visual disturbance, and cranial nerve palsy. One case in Table 1 presented with altered consciousness as our case did. Nawar et al. reported 11 cases with hemorrhage within RCC [14]. In their study, although not described in detail of each case, respectively, there were 3 patients with altered consciousness, there were 10 patients with headache, there were 3 patients with a visual deficit, and there was 1 patient with cranial nerve palsy.

The pituitary apoplexy is caused by the anticoagulant therapy, the bromocriptine therapy, the radiation therapy, the hormone loading test, the cerebral angiography, and the cardiovascular surgery [18]. Particularly in macroadenoma, the head trauma can cause the apoplexy between 0 days and 21 days [19–21]. None of these reviewed cases of RCC apoplexy were caused by the head trauma. In our case, it was unclear whether the unconsciousness at the first emergency visit depended on the head trauma or apoplexy.

Preoperative endocrinological evaluations revealed abnormalities in 4 cases (Table 1). Wakai et al. described that preoperative endocrinological examinations revealed abnormalities in 3 cases. Most pituitary apoplexy cases need hormone replacement therapy for partial hypopituitarism [6]. In our case the preoperative endocrinological examinations were normal.

A rare complication of pituitary apoplexy is cerebral infarction, which is caused by either direct compression of an artery or vasospasm. Twenty cases of pituitary apoplexy followed by cerebral infarction have been reported in the literature (Table 2) [1, 20, 22–24, 26, 28, 29, 31–39]. The ischemic events were attributed to mechanical compression by the tumor in 12 cases and to cerebral vasospasm in 8 cases. The cerebral infarction was located at the anterior cerebral artery (ACA) territory in 4 cases, the MCA territory in 8 cases, and the ICA territory in 4 cases. Cerebral arterial stenosis was detected in the ICA in 15 cases, in the MCA in 3 cases, and in the ACA in 3 cases. The cerebral infarction occurred between 0 and 21 days after the onset of pituitary apoplexy. Most infarctions due to cerebral vasospasm occurred between 5 and 21 days after onset, and all infarctions by mechanical compression occurred within 2 days after onset.

In our case, the cerebral infarction occurred in the right MCA territory after a 13-day interval from the onset of RCC apoplexy. MRA revealed the stenotic lesion of the right MCA, and echocardiogram, electrocardiogram, and carotid artery ultrasound showed no abnormalities. These clinical findings could exclude the embolic cause of the ischemic lesion. Our case was neither hyperlipidemic nor hypertensive. CTA and carotid artery ultrasound presented no artherosclerotic changes. These clinical findings could exclude the possibility of the artherosclerotic brain infarction. Postoperative MRA and CTA showed that the visibility and stenosis of right MCAs were recovered. Therefore together these findings suggested that the etiology of the cerebral infarction was cerebral vasospasm.

The pathology of vasospasm following pituitary apoplexy is unclear; however, vasoactive chemical substances released during pituitary apoplexy may cause vasospasm [20, 28, 35]. The subarachnoid hemorrhage (SAH) causes the vasospasm, but the pathological mechanism also remains unclear [27]. The endothelin, angiotensin, and sphingosine metabolites are one of the vasoactive substances, respectively [5, 25, 30]. Although the CT and MRI detected no SAH in our case, vasoactive substances liberated from the hemorrhagic RCC or SAH below levels in detection by CT and MRI might cause the vasospasm.

This patient had fallen from her motorbike. She was disoriented and had a facial abrasion. Considering head trauma, the attending physician examined head CT. This physician overlooked a significant appearance in the suprasellar lesion. Therefore the attending physician did not consult a neurosurgeon.

Asymptomatic incidental RCC should be monitored conservatively. Symptomatic RCC can benefit from surgical decompression, and RCC apoplexy should be considered the surgical decompression to prevent cerebral infarction, even if symptoms resolve soon after onset.

## Figures and Tables

**Table 1:** Summary of the clinical presentations and endocrinological findings in reported cases of RCC apoplexy.

Author and year	Age, sex	Presentation	Preop endocrine findings	Postop endocrine results
Onesti et al., 1990 [16]	25, F	Headache, nausea	Normal	Normal
Kleinschmidt-DeMasters et al., 1995 [11]	51, F	Visual deterioration	NA	NA
Kurisaka et al., 1998 [18]	8, F	Headache	Normal	Normal
Nishioka et al., 1999 [15]	46, F	Headache, visual loss, nausea	Normal	Normal
Fukushima et al., 2001 [22]	67, F	Headache, nausea, ptosis	Normal	HRT for cortisol
Pawar et al., 2002 [17]	19, M	Headache, blurred vision	Normal	Normal
Rosales et al., 2004 [23]	34, M	Headache, diplopia	PRL elevation, decreased T4	HRT for DI and thyroid
Binning et al., 2008 [9]	24, F	Headache	Normal	Normal
	20, M	Headache, nausea, diplopia	Decreased testosterone	HRT for testosterone
	23, F	Headache, visual loss	PRL elevation	Normal
	49, M	Headache	Normal	Normal
	21, F	Headache	Decreased T4	HRT for thyroid
	54, F	Headache, visual loss	Normal	Normal
Raper and Besser, 2009 [24]	72, F	NA	NA	NA
Present case	67, F	Hemiparesis	Normal	Normal

PRL, prolactin; DI, diabetes insipidus; HRT, hormone replacement therapy; NA, not available.

**Table 2:** Reported cases of cerebral ischemia following pituitary apoplexy.

Author and year	Age, sex	Territory of infarction	Angiographical findings	Symptom	Days after onset	Mechanism	Pathology
Rosenbaum et al., 1977 [25]	77, M	Right MCA	Right ICA occlusion, left ICA stenosis	Left hemiparesis	0	c	PA
Cardoso and Peterson, 1983 [19]	34, F	Diffuse	Bil. ICA, ACA, MCA stenosis	Reduced consciousness	21	v	PA
	38, M	NA	Bil. ICA, BA stenosis	Lethargic	0	v	PA
Bernstein et al., 1984 [8]	48, M	NA	Bil. ICA stenosis	Reduced consciousness, hemiparesis	0	c	PA
Clark et al., 1987 [26]	40, M	Left ACA	Right ICA stenosis, left ICA occlusion	Dysphasia, right hemianopia, right hemiparesis	0	c	PT
Pozzati et al., 1987 [21]	15, M	Right MCA	Bil. ICA stenosis	Reduced consciousness	0	v	PT
Itoyama et al., 1990 [27]	45, M	NA	Left ICA, MCA stenosis	Reduced consciousness, right hemiparesis	14	v	PA
Yaghmai et al., 1996 [28]	47, M	None	Right ICA occlusion	Right blindness	1	c	PA
Lath and Rajshekhar, 2001 [12]	40, M	Right ICA	Right ICA occlusion	Left hemiparesis	1	c	PA
Rodier et al., 2003 [29]	35, M	Right ICA, ACA	Bil. ACA, right MCA stenosis	Reduced consciousness, left hemiparesis	2	c and v	PA
Akutsu et al., 2004 [30]	29, M	Left MCA	Left ACA stenosis	Reduced consciousness, right hemiparesis	5	v	PA
Byung et al., 2007 [10]	41, M	Left MCA	NA	Right hemiparesis, dysarthria	7	v	PA
Dogan et al., 2008 [31]	50, M	Left ICA	Left ICA occlusion	Reduced consciousness	0	c	PA
Das et al., 2008 [32]	46, M	Left MCA	Left ICA stenosis	Right hemiparesis	0	c	PA
Ahmed and Semple, 2008 [33]	51, M	Bil. ACA	NA	Left hemiparesis	0	c	PA
	31, F	Bil. ACA	NA	Bil. blindness, reduced consciousness	14	v	PA
Yang et al., 2008 [34]	43, M	Right ICA	Right ICA stenosis	Left hemiparesis	0	c	PA
López Hernández, 2008 [35]	23, M	—	—	—	—	—	PT
Lill et al., 2009 [13]	59, M	Bil. ICA	Bil. ICA occlusion	Left hemiparesis, reduced consciousness	0	c	PA
Chokyu et al., 2011 [36]	50, M	Right MCA	Right ICA occlusion	Left hemiparesis	0	c	PA
Present case	67, F	Right MCA	Right MCA stenosis	Reduced consciousness, left hemiparesis	13	v	RCC

ACA, anterior cerebral artery; MCA, middle cerebral artery; ICA, internal carotid artery; c, compression; v, vasospasm; PA, pituitary adenoma; PT, pituitary tumor.
